# Ridge regression and its applications in genetic studies

**DOI:** 10.1371/journal.pone.0245376

**Published:** 2021-04-08

**Authors:** M. Arashi, M. Roozbeh, N. A. Hamzah, M. Gasparini

**Affiliations:** 1 Department of Statistics, Faculty of Mathematical Sciences, Ferdowsi University of Mashhad, Mashhad, Iran; 2 Department of Statistics, Faculty of Mathematics, Statistics and Computer Sciences, Semnan University, Semnan, Iran; 3 UM Centre of Data Analytics, Institute of Mathematical Sciences, University of Malaya, Kuala Lumpur, Malaysia; 4 Faculty of Mathematics, Polytechnic of Torino University, Torino, Italy; Roswell Park Cancer Institute, UNITED STATES

## Abstract

With the advancement of technology, analysis of large-scale data of gene expression is feasible and has become very popular in the era of machine learning. This paper develops an improved ridge approach for the genome regression modeling. When multicollinearity exists in the data set with outliers, we consider a robust ridge estimator, namely the rank ridge regression estimator, for parameter estimation and prediction. On the other hand, the efficiency of the rank ridge regression estimator is highly dependent on the ridge parameter. In general, it is difficult to provide a satisfactory answer about the selection for the ridge parameter. Because of the good properties of generalized cross validation (GCV) and its simplicity, we use it to choose the optimum value of the ridge parameter. The GCV function creates a balance between the precision of the estimators and the bias caused by the ridge estimation. It behaves like an improved estimator of risk and can be used when the number of explanatory variables is larger than the sample size in high-dimensional problems. Finally, some numerical illustrations are given to support our findings.

## Introduction

High-dimensional statistical inference is essential whenever the number of unknown parameters is larger than sample size. Typically, high-throughput technology provides large-scale data of gene expressions in transcriptomics. As an example, the riboflavin production data set with Bacillus subtilis (Lee *et al.* [1] and Zamboni *et al.* [2]) includes the logarithm of the riboflavin production rate as the response variable along with 4088 covariates which are the logarithm of the expression levels of 4088 genes, which are normalized using the Affymetrix oligonucleotide arrays normalizing methods. One rather homogeneous data set exists from 71 samples that were hybridized repeatedly during a fed-batch fermentation process in which different engineered strains and strains grown under different fermentation conditions were analyzed.

A relevant family of methods for prediction of the response based on the high dimensional gene expression data are sparse linear regression models. The least absolute shrinkage and selection operator (LASSO), proposed by Tibshirani [3], is the most popular, while other relevant methods are SCAD penalization [4] and minimum concave penalty [5]. In spite of the suitable sparsity caused by these penalized methods, they have low prediction performance for high dimensional data sets, because of their shrinkage and bias. Hence, developing shrinkage strategies to improve prediction is an interest in genome studies.

**Fig 1:**
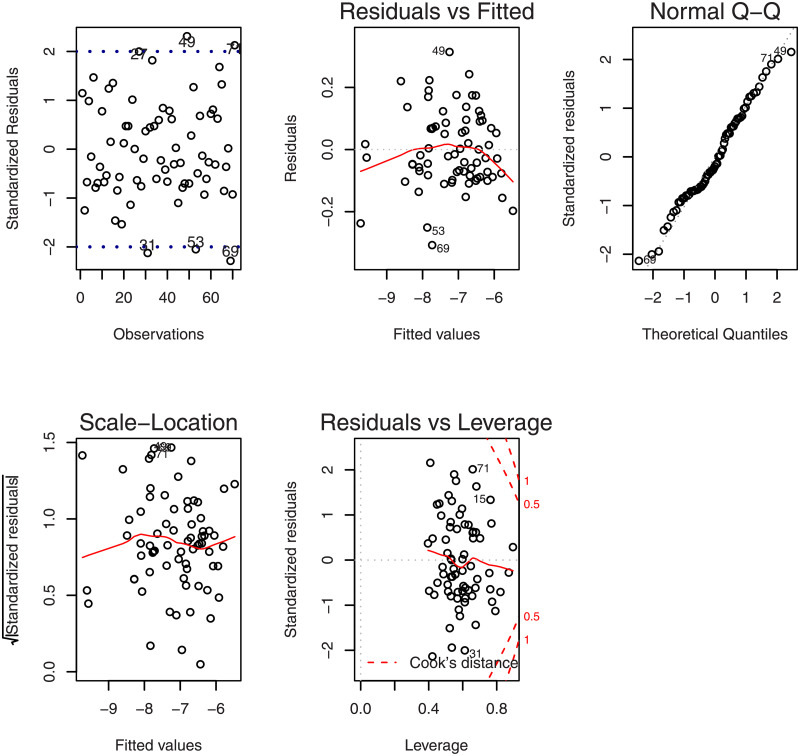
Diagnostic plots for the riboflavin production data set.

The primary aim of this study is improving the prediction accuracy of the riboflavin production data, in genome regression modeling; secondly, we further focus on detecting outliers. Intuitive methods for labeling observations as outliers can be provided by diagnostic plots. Fig 1 gives the diagnostics plots to identify outliers for the riboflavin data set based on the ordinary least-squares model with effective genes. The plots suggest there exist some outliers in the data set. Hence, developing efficient robust estimation strategy is another aspect of our approach.

### Rank regression

Jureckova [6] and Jaeckel [7] proposed rank-based estimators for linear models as highly efficient and robust methods to outliers in response space. In short, rank regression is a simple technique which consists of replacing the data with their corresponding ranks. Rank regression and related inferential methods are useful in situations where

1. the relation between the response and covariate variables is nonlinear and monotonic and a simple and practical nonlinear form is of interest rather than polynomial, spline, kernel and/or other forms
2. there are outliers present in the study and we need a nonparametric robust procedure
3. the mere presence of so many important input variables makes it difficult to think in terms to find an appropriate parametric nonlinear model

The package Rfit in R, developed by Kloke and McKean [8] is a convenient and efficient tool for estimation/testing, diagnostic procedures and measures of influential cases in rank regression.

### Rank estimator

Consider the setting where observed data are realizations of ${\left\{\left(X_{i},y_{i}\right)\right\}}_{i=1}^{n}$ with *p*-dimensional covariates ***X***_*i*_ ∈ **R**^*p*^ and univariate continuous response variables *y*_*i*_ ∈ **R**. A simple regression model has form

$$\begin{array}{c} y_{i}=X_{i}^{⊤}\beta +\epsilon_{i}, \end{array}$$ (1)

where ***β*** is the vector of regression coefficients and *ϵ*_*i*_ is the *i*^*th*^ error component. For simplicity, we assume that the intercept is zero. In case it exists, by centering the observations one can eliminate it from the study.

We assume that:

1. Errors ***ϵ*** = (*ϵ*_1_, …, *ϵ*_*n*_)^⊤^ are independently and identically distributed (i.i.d.) random variables with (unknown) cumulative distribution function (c.d.f.) *F* having absolutely continuous probability density function (p.d.f.) *f* with finite and nonzero Fisher information
  $$\begin{array}{c} 0<I\left(f\right)=\int_{-\infty }^{+\infty }{\left[-\frac{f^{\prime }\left(x\right)}{f(x)}\right]}^{2}f\left(x\right)dx<\infty . \end{array}$$
2. For obtaining the linear rank estimator, we consider the score generating function ψ:(0,1)→R which is assumed to be non constant, nondecreasing, and square integrable on (0, 1). The scores are defined in either of the following ways:
  $$\begin{array}{c} a\left(i\right)=E\psi \left(U_{i:n}\right)\;\text{or}\;a\left(i\right)=\psi (\frac{i}{n+1}),\;i=1,\ldots ,n \end{array}$$
  for *n* ≥ 1, where *U*_1:*n*_ ≤ … ≤ *U*_*n*: *n*_ are order statistics from a sample of size *n* from the uniform distribution U(0,1).

To obtain the rank estimate of ***β***, define the pseudo-norm

$$\begin{array}{c} {∥v∥}_{\psi }=\sum_{i=1}^{n}a\left(R\left(v_{i}\right)\right)v_{i} \end{array}$$ (2)

where for ***y*** = (*y*_1_, …, *y*_*n*_)^⊤^, ***a***(*R*(***y***)) = (*a*(*R*(*y*_1_)), …, *a*(*R*(*y*_*n*_)))^⊤^ and *R*(*y*_*i*_) is the rank of *y*_*i*_, *i* = 1, …, *n* and *a*(1)≤*a*(2)≤…≤*a*(*n*). Then, the rank-estimate of ***β*** is given by

$$\begin{array}{ll} {\hat{\beta }}_{\psi } & =\text{arg}\;\text{min}∥y-X\beta {∥}_{\psi }\\ & ={\left(X^{⊤}X\right)}^{-1}X^{⊤}{\hat{y}}_{\psi }, \end{array}$$ (3)

where ***X*** = (***X***_1_, …, ***X***_*n*_)^⊤^ and ${\hat{y}}_{\psi }$ is the minimizer of dispersion function *D*_*ψ*_(**η**) = ‖***y*** − **η**‖_*ψ*_ over ηϵC(X), where C(X) is the column space spanned by the columns of ***X***. Thus, ${\hat{\beta }}_{\psi }$ is the solution to the rank-normal equations ***X***^⊤^ ***a***(*R*(***y*** − ***Xβ***)) = **0** and $D_{\psi }\left(X{\hat{\beta }}_{\psi }\right)={∥y-{\hat{y}}_{\psi }∥}_{\psi }$. Refer to the “S1 File” for a simple example of rank estimator, the form of *ψ*, and references.

### Regularization methods

Under situations in which the matrix ***X***^⊤^ ***X*** is singular, the usual estimators are not applicable. From a practical point of view, high empirical correlations among two or a few other covariates lead to unstable results for estimating ***β*** or for pursuing variable selection. To overcome this problem, the ridge version for estimation can be considered. Ridge estimation is a regularization technique initially introduced by Tikhonov [9] and followed by Hoerl and Kennard [10] to regularize parameters or linear combination of them in order to provide acceptable estimators with less variability than the usual estimator, in multicollinear situations (see [8, 11–14] for more details). On the other hand, when the response distribution is non-normal or there are some outliers present in the study, the usual least squares and maximum likelihood methods fail to provide efficient estimates. In such cases, there is a need to develop an estimation strategy which is applicable in multicollinear situations and has acceptable efficiency in the presence of outliers.

### Our contribution

Our contribution is two fold. First, for the situations where both multicollinearity and outliers exist we develop a shrinkage estimation strategy based on the rank ridge regression estimator. This creates two tuning parameters that must be optimized. Then, we define a new generalized cross validation (GCV) criterion to select the induced tuning parameters. The GCV has been applied to obtain the optimal ridge parameter in a ridge regression model by Golub *et al.* [15] and to obtain the optimal ridge parameter and bandwidth of the kernel smoother in semiparametric regression model by Amini and Roozbeh [16] as well as in partial linear models by Speckman [17]. Here, we use the GCV criterion for selecting the optimal values of ridge and shrinkage parameters, simultaneously. Our proposed GCV criterion creates a balance between the precision of the estimators and the biasedness caused by the ridge and shrinkage parameters.

The following section provides a robust shrinkage estimator based on the improved rank-based test statistic with developing a generalized cross validation (GCV) criterion, to obtain optimal values of tuning parameters. Subsequently, application of the proposed improved estimation method is illustrated for two real world data sets and an extensive simulation study to demonstrate usefulness of the proposed improved methodology. Finally, our study is concluded.

## Methodology

In this section, we first define a robust test-statistic for testing the null hypothesis $H_{o}:\beta =0$ in the rank-regression analysis. This test is further employed in the construction of a robust rank-based shrinkage estimator. Then, we consider the rank ridge regression estimator and by the aid of the proposed test-statistic, we define the Stein-type shrinkage estimator for ***β*** for robust analysis. Since this estimator will have two tuning parameters, we evaluate these parameters using a generalized cross validation (GCV) criterion.

### Robust shrinkage estimator

In order to define the robust rank-based shrinkage estimator, we need to develop a robust test statistic to test the following set of hypotheses

$$\begin{array}{c} H_{o}:\beta =0\;vs\;H_{A}:\beta \neq 0. \end{array}$$ (4)

Denote the *i*^*th*^ element of ***H*** = ***X***(***X***^⊤^ ***X***)^−1^ ***X***^⊤^, the projection matrix onto the space C(X), by *h*_*iin*_. We also need the following regularity conditions to be held.

(A1) lim_*n* → ∞_max_1≤*i*≤*n*_ *h*_*iin*_ = 0, where *h*_*iin*_ is commonly called the leverage of the *i*^*th*^ data point.
(A2) $\lim_{n\to \infty }\frac{1}{n}X^{⊤}X=\Sigma$, where **Σ** is a *p* × *p* positive definite matrix.

**Theorem 1** *Let*

$$\begin{array}{c} R_{n}\left(k\right)=\sigma_{a}^{-2}a^{⊤}\left(R\left(y\right)\right)X{\left(n^{-1}X^{⊤}X+kI_{p}\right)}^{-1}{\left[X\left(k\right)\right]}^{-1}{\left(n^{-1}X^{⊤}X+kI_{p}\right)}^{-1}X^{⊤}a\left(R\left(y\right)\right), \end{array}$$ (5)

where ***X***(*k*) is an invertible matrix given by

$$\begin{array}{c} X\left(k\right)={\left(\frac{1}{n}X^{⊤}X+I_{p}\right)}^{-1}-[k{\left(\frac{1}{n}X^{⊤}X+I_{p}\right)}^{-1}{\left(\frac{1}{n}X^{⊤}X+I_{p}\right)}^{-1}], \end{array}$$ (6)

$\sigma_{a}^{2}=\frac{1}{n-1}\sum_{j=1}^{n}a^{2}\left(j\right)≐1$, *and k* > 0. *Assume (A1) and (A2). Then, reject* $H_{o}$ *in favor of* $H_{A}$ *at approximate level α iff* $R_{n}\left(k\right)\geq \chi_{p}^{2}\left(\alpha \right)$, *where* $\chi_{p}^{2}\left(\alpha \right)$ *denotes the upper level α critical value of χ^2^ distribution with *p* d.f*.

**Proof 1** *Refer to the* “S1 File”.

Now, using a similar approach in formulating the ridge estimator, we use the following rank ridge regression estimator (Roozbeh *et al.* [18])

$$\begin{array}{ccc} {\hat{\beta }}_{\psi }\left(k\right) & = & {\left(\frac{1}{n}X^{⊤}X+kI_{p}\right)}^{-1}X^{⊤}{\hat{y}}_{\psi }, \end{array}$$ (7)

where *k* > 0 is the ridge parameter.

In order to improve upon the rank ridge regression estimator, following Saleh [19], we use the Stein-type shrinkage estimator (SSE) as

$$\begin{array}{ll} {\hat{\beta }}_{\psi }^{\left(S\right)}(k,d) & =(1-\frac{d}{R_{n}(k)}){\hat{\beta }}_{\psi }(k)\\ & ={\hat{\beta }}_{\psi }(k)-dR_{n}{\left(k\right)}^{-1}{\hat{\beta }}_{\psi }(k),d>0. \end{array}$$ (8)

The SSE shrinks the coefficients towards the origin using the test statistic *R*_*n*_(*k*). The amount of shrinkage is controlled by the shrinkage coefficient *d*.

In the following result we show that the SSE is a shrinkage estimator with respect to the *l*_*q*_-norm, ${∥a∥}^{q}={\left(\sum_{j=1}^{n}{\left|a_{j}\right|}^{q}\right)}^{1/q}$, *q* > 0, with ***a*** = (*a*_1_, …, *a*_*n*_)^⊤^. The reason we take the *l*_*q*_-norm is that we can simultaneously take *l*_1_ and *l*_2_ norms into consideration. One must consider *l*_1_-norm keeps the scale of observation, however, *l*_2_-norm is mathematically tractable.

**Theorem 2** ${\hat{\beta }}_{\psi }^{\left(S\right)}\left(k,d\right)$ *is a shrinkage estimator under l*_*q*_-*norm under some regularity conditions as stated below*

*(i): Under the set of local alternatives* $K_{n}:\beta =n^{-\frac{1}{2}}\;\delta$, *with* **δ** = (*δ*_1_, …, *δ*_*p*_)^⊤^, *δ*_*i*_ ≠ 0, *i* = 1, …, *p*, *we have* $∥{\hat{\beta }}_{\psi }^{\left(S\right)}{\left(k,d\right)∥}^{q}<{∥{\hat{\beta }}_{\psi }\left(k\right)∥}^{q}$.
*(ii) For k* > *n*/2, *d* > 0, *we have*
  $$\begin{array}{c} ∥{\hat{\beta }}_{\psi }^{\left(S\right)}{\left(k,d\right)∥}^{q}<{∥(1-\frac{d}{R_{n}\left(k\right)}){\hat{y}}_{\psi }∥}^{q}. \end{array}$$
*(iii) Assume λ*_*i*_ = *o*(*n*), *i* = 1, …, *n*. *For k* > sup_1≤*i* ≤ *n*_ *λ*_*i*_, *(ii) holds in limit*.

**Proof 2** Refer to the “S1 File”.

The proposed SSE may be criticized since it depends on the two tuning parameters *k* and *d* and it may come to mind why we need an estimator with two tuning parameters, when we have the rank ridge regression estimator. In what follows we elaborate more on the advantages of the SSE ${\hat{\beta }}_{\psi }^{\left(S\right)}\left(k,d\right)$ in our analysis. Accepting the fact that we need a robust rank estimator, apart from the justifications provided in Saleh [19], we give the following reasons.

1. Apparently, as *d* → 0, ${\hat{\beta }}_{\psi }^{\left(S\right)}\left(k,d\right)\to {\hat{\beta }}_{\psi }\left(k\right)$ and thus for small values *d* the gain in estimation is just the information provided by the robust ridge parameter, even if the null hypothesis $H_{o}$ is not true. Thus, even if we agree that the rank ridge regression estimator shrink the coefficients to zero, the information provided by the test statistic *R*_*n*_(*k*), which is controlled by *d* in the SSE, is useful.
2. Consider a situation in which we do not have strong evidence to reject the null hypothesis. Knowing the fact that the ridge estimator does not select variables (see Saleh *et al.* [20]), we can not estimate the zero vector using the rank ridge regression estimator, however, the shrinkage coefficient *d* maybe obtained such that for a given *k*, *d* = *R*_*n*_(*k*) and the resulting shrinkage estimator becomes equal to zero. This might be a rare event, but theoretically sounds.
3. The last but not the least, for the set of local alternatives $K_{n}$, as in Theorem 2, the proposed SSE shrinks more than the rank ridge regression estimator. Thus in order to have robust shrinkage estimator, the SSE with two tuning parameters is preferred.

The SSE depends on both the ridge parameter *k* and shrinkage parameter *d*. For optimization purposes, we use the GCV of Roozbeh *et al.* [18] in the forthcoming section.

## Generalized cross validation

The GCV chooses the ridge and shrinkage parameters by minimizing an estimate of the unobservable risk function

$$\begin{array}{ccc} R(\beta ;{\hat{\beta }}_{\psi }^{\left(S\right)}\left(k,d\right)) & = & \frac{1}{n}{\left(X\beta -{\hat{y}}_{\psi }^{\left(S\right)}\left(k,d\right)\right)}^{⊤}(X\beta -{\hat{y}}_{\psi }^{\left(S\right)}\left(k,d\right))\\ & = & \frac{1}{n}{∥X\beta -X{\hat{\beta }}_{\psi }^{\left(S\right)}\left(k,d\right)∥}^{2}, \end{array}$$

where

$$\begin{array}{ccc} {\hat{y}}_{\psi }^{\left(S\right)}\left(k,d\right) & = & X{\hat{\beta }}_{\psi }^{\left(S\right)}\left(k,d\right)\\ & = & (1-\frac{d}{R_{n}\left(k\right)})2{\hat{\tau }}_{\psi }X{\left(n^{-1}X^{⊤}X+kI_{p}\right)}^{-1}X^{⊤}y\\ & = & L(k,d)y, \end{array}$$ (9)

with $L\left(k,d\right)=(1-\frac{d}{R_{n}\left(k\right)})2{\hat{\tau }}_{\psi }X{\left(n^{-1}X^{⊤}X+kI_{p}\right)}^{-1}X^{⊤}$, termed as the hat matrix of y, and ${\hat{\tau }}_{\psi }$ is a consistent estimator (see Hettmansperger and McKean [21]) for the scale parameter *τ*_*ψ*_ given by

$$\begin{array}{c} \tau_{\psi }^{-1}=\int \psi \left(u\right)\psi_{f}\left(u\right)du,\;\psi_{f}\left(u\right)=-\frac{f^{\prime }\left(F^{-1}\left(u\right)\right)}{f(F^{-1}\left(u\right))}. \end{array}$$

It is straightforward to show that (see Hettmansperger and McKean [21] and Roozbeh [22])

$$\begin{array}{ccc} E(R(\beta ;{\hat{\beta }}_{\psi }^{\left(S\right)}\left(k,d\right))) & = & \frac{1}{n}∥(I_{n}-L\left(k,d\right))X\beta {∥}^{2}+\frac{\tau_{\psi }^{2}}{n}\text{tr}(L{\left(k,d\right)}^{2})\\ & = & b^{2}\left(k,d\right)+\tau_{\psi }^{2}\mu_{2}\left(k,d\right), \end{array}$$

where $b^{2}\left(k,d\right)=\frac{1}{n}∥(I_{n}-L\left(k,d\right))X\beta {∥}^{2}$ and $\mu_{2}\left(k,d\right)=\frac{1}{n}\text{tr}(L{\left(k,d\right)}^{2})$.

The GCV function is then defined as

$$\begin{array}{ccc} \text{GCV}({\hat{\beta }}_{\psi }^{\left(S\right)}\left(k,d\right)) & = & \frac{\frac{1}{n}∥(I_{n}-L\left(k,d\right))y{∥}^{2}}{{\left(1-\frac{1}{n}\text{tr}(L(k,d))\right)}^{2}}\\ & = & \frac{\frac{1}{n}∥(I_{n}-L\left(k,d\right))y{∥}^{2}}{{\left(1-\mu_{1}\left(k,d\right)\right)}^{2}}, \end{array}$$ (10)

where $\mu_{1}\left(k,d\right)=\frac{1}{n}\text{tr}(L(k,d))$.

**Corollary 3** *Suppose that the eigenvalues* {*λ*_*νn*_, *ν* = 1, …, *n*} of ***XX***^⊤^ *satisfy λ*_*νn*_ ≃ *nν*^−*m*^ *for some m* > 1. *Then, for the GCV function in* (10)

$$\begin{array}{ccc} \lim_{n\to \infty }E(\text{GCV}({\hat{\beta }}_{\psi }^{\left(S\right)}\left(k,d\right))) & = & \tau_{\psi }^{2}+\lim_{n\to \infty }E(R(\beta ;{\hat{\beta }}_{\psi }^{\left(S\right)}\left(k,d\right))). \end{array}$$ (11)

Corollary 3 is an application of the GCV theorem of Craven and Wahba (1979) and Golub *et al.* (1979). It implies that the minimizer of $E(\text{GCV}({\hat{\beta }}_{\psi }^{\left(S\right)}\left(k,d\right)))$ is essentially equivalent to the minimizer of $E(R(\beta ;{\hat{\beta }}_{\psi }^{\left(S\right)}\left(k,d\right)))$ for SSE. Based on (11), $\text{GCV}({\hat{\beta }}_{\psi }^{\left(S\right)}\left(k,d\right))$ is an estimator of $E(R(\beta ;{\hat{\beta }}_{\psi }^{\left(S\right)}\left(k,d\right)))$ with a nearly constant bias. Using the techniques of Section 3, this can be shown to be an estimator of $\tau_{\psi }^{2}$ with positive but asymptotically negligible bias, so the resulting “F” statistic can be expected to be conservative. The main result of this section is to obtain a good estimate of the minimizer of $E(R(\beta ;{\hat{\beta }}_{\psi }^{\left(S\right)}\left(k,d\right)))$ from the data which does not require knowledge of $\tau_{\psi }^{2}$ so that, by minimizing it, we can extract the optimal values for the two tuning parameters simultaneously.

## Applications

In this section we consider some numerical experiments to illustrate the usefulness of the suggested improved methodology in the regression model. We analyze the performance of the proposed estimators in a real-world examples related to the riboflavin production.

### Application to riboflavin production data set

To support our assertions, we consider the data set about riboflavin (vitamin B2) production in Bacillus subtilis, which can be found in R package “hdi”. There is a single real valued response variable which is the logarithm of the riboflavin production rate and *p* = 4088 explanatory variables measuring the logarithm of the expression level of 4088 genes. There is one rather homogeneous data set from *n* = 71 samples that were hybridized repeatedly during a fed batch fermentation process where different engineered strains and strains grown under different fermentation conditions were analyzed.

**Fig 2:**
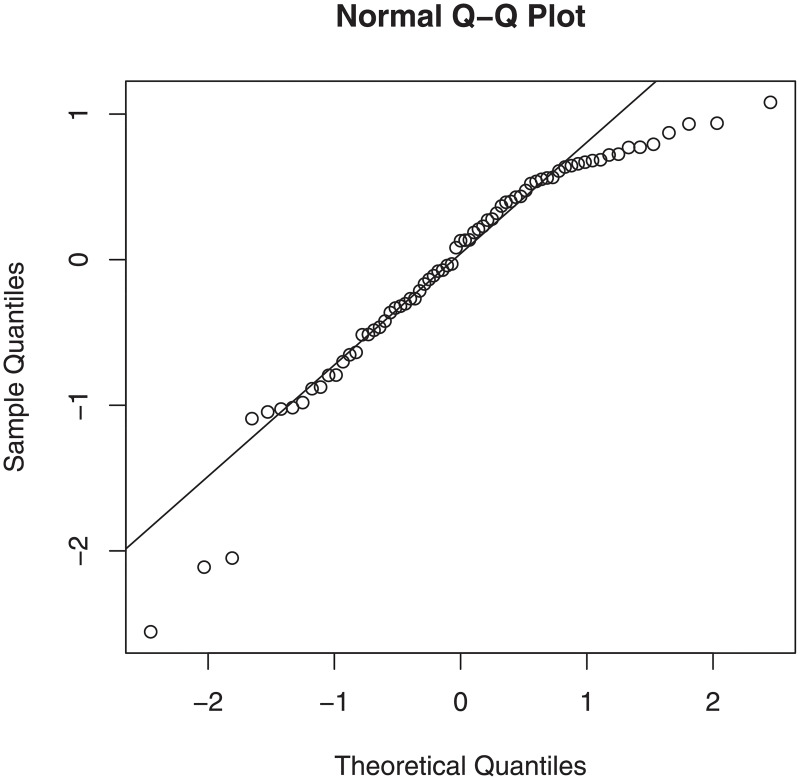
Q–Q plot based on the ridge estimator for the riboflavin production data set.

**Fig 3:**
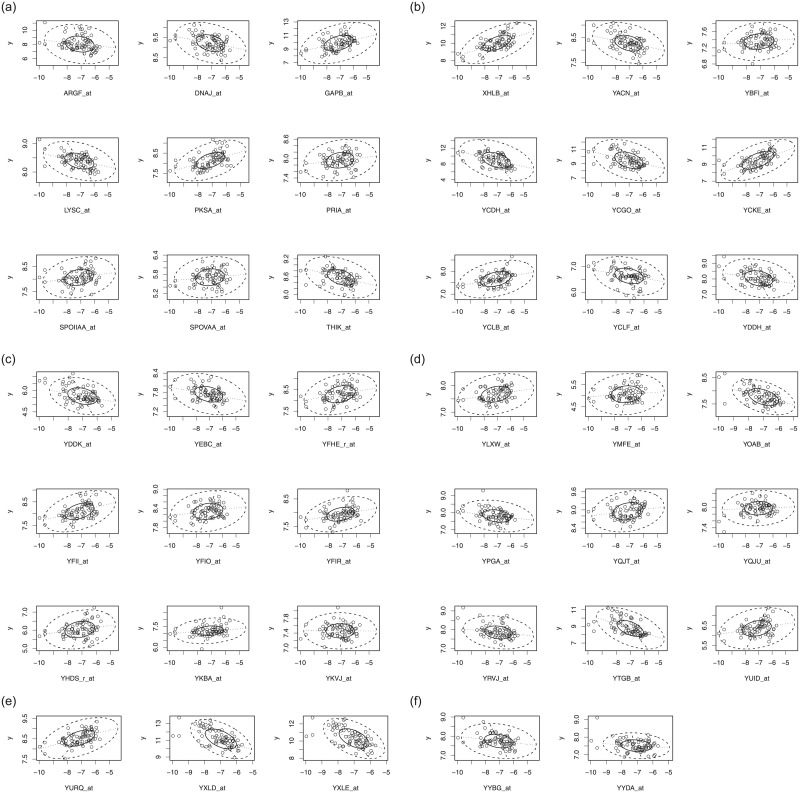
Bivariate boxplot of the riboflavin production data set for effective genes.

Fig 2 shows the normal Q–Q plot based on the ridge estimation for the riboflavin production data set. Also, the bivariate boxplot for selected genes of this data is depicted in Fig 3. The bivariate boxplot is a two-dimensional analogue of the boxplot for univariate data. This diagram is based on calculating robust measures of location, scale, and correlation; it consists essentially a pair of concentric ellipses, one of which (the hinge) includes 50% of the data and the other (called the fence) delineates potentially troublesome outliers. In addition, robust regression lines of both response on predictor and vice versa are shown, with their intersection showing the bivariate location estimator. The acute (large) angle between the regression lines will be small (large) for a large (small) absolute value of correlations. Figs 2 and 3 clearly reveals that the data contains some outliers.

We use GCV to select the the ridge and shrinkage parameters for the proposed estimators, simultaneously. Similar to the SSE, the GCV score functions for $\hat{\beta }\left(k\right)$ and ${\hat{\beta }}_{\psi }\left(k\right)$ can be procured by setting

$$\begin{array}{c} L_{1}\left(k\right)=X{\left(n^{-1}X^{⊤}X+kI_{p}\right)}^{-1}X^{⊤},L_{2}\left(k\right)=2{\hat{\tau }}_{\psi }X{\left(n^{-1}X^{⊤}X+kI_{p}\right)}^{-1}X^{⊤}, \end{array}$$

in (10), respectively.

**Fig 4:**
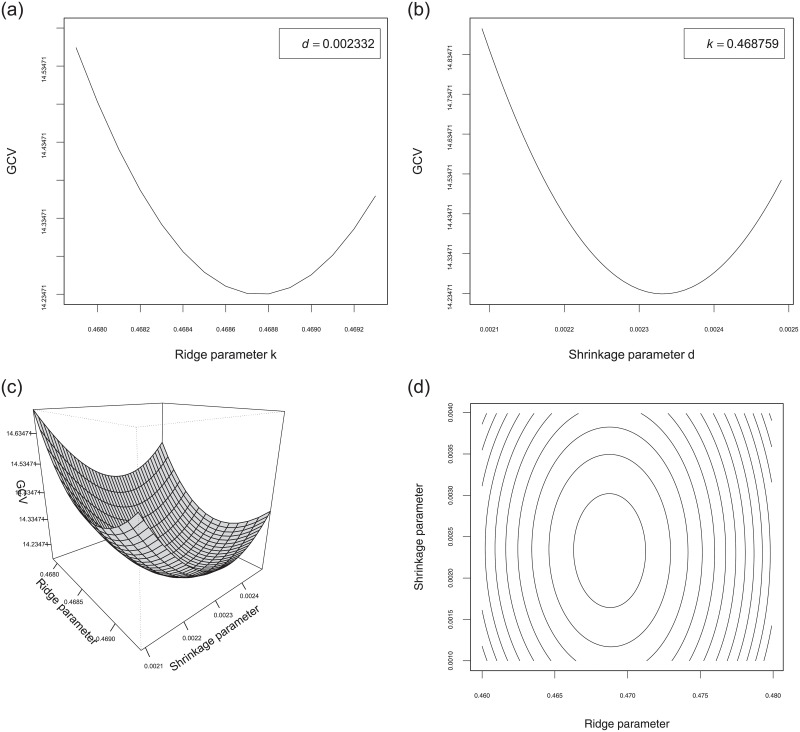
The diagram of $\text{GCV}({\hat{\beta }}_{\psi }^{\left(S\right)}\left(k,d\right))$ and its counter plot versus *k* and *d* for the riboflavin production data set.

All computations were conducted using the statistical software R 3.4.3 to develop the package Rfit for calculating the proposed estimators, their test statistics and powers described in this paper. The R codes are available at https://mahdiroozbeh.profile.semnan.ac.ir/#downloads. The 3D diagram as well as the 2D slices of GCV of ${\hat{\beta }}_{\psi }^{\left(S\right)}\left(k,d\right)$ versus *k* and *d* are plotted in Fig 4 for the riboflavin production data set. As it can be seen from Fig 4, the 2D (3D) diagrams of GCV are convex functions (surfaces) and hence they have a global minimum. This guarantees the existence of optimum values of *k* and *d* which minimize the GCV’s. The minimum of $\text{GCV}({\hat{\beta }}_{\psi }^{\left(S\right)}\left(k,d\right))$ approximately occurs at *k*_*opt*_ = 0.468759 and *d*_*opt*_ = 0.002332. We test the hypothesis $H_{o}:\beta =0$ using the ridge rank-based (RRB) test statistic. The test statistic for $H_{o}$, given our observations, is *R*_*n*_(*k*_*opt*_) = 27.42. Thus, we conclude that there is not enough evidence to reject the null hypothesis $H_{o}$ and so, the SSE can be efficient for prediction purposes according to the second comment right after Theorem 2.

To measure the prediction accuracy of proposed estimators, the leave-one-out cross-validation (CV) criterion was used, which is defined by

$$\begin{array}{c} \text{CV}\left(\hat{\beta }\right)=\frac{1}{n}\sum_{s=1}^{n}{\left(y_{\left(-s\right)}-X_{\left(-s\right)}{\hat{\beta }}_{\left(-s\right)}\right)}^{2}, \end{array}$$

where ${\hat{\beta }}_{\left(-s\right)}$ is obtained by replacing X and y with $X_{\left(-s\right)}=({\tilde{x}}_{jk(-s)})$, 1 ≤ *k* ≤ *n*, 1 ≤ *j* ≤ *p*, $\;y_{\left(-s\right)}={\left({\tilde{y}}_{1(-s)},\ldots ,{\tilde{y}}_{n(-s)}\right)}^{⊤}$, ${\tilde{x}}_{lk(-s)}=x_{lk}-\sum_{j\neq s}^{n}W_{nj}\left(t_{s}\right)x_{lj}$, ${\tilde{y}}_{k(-s)}=y_{k}-\sum_{j\neq s}^{n}W_{nj}\left(t_{s}\right)y_{j}$. Here $y_{\left(-s\right)}$ is the predicted value of response variable where *s*th observation left out of the estimation of the ***β***.

Table 1 displays a summary of the results. In this Table, a goodness of fit criterion R-squared is calculated for comparing the proposed estimators using the following formula

$$\begin{array}{c} R^{2}\left(\hat{\beta }\right)=1-\frac{\text{SSE}(\hat{\beta })}{S_{yy}}, \end{array}$$

where $\text{SSE}\left(\hat{\beta }\right)=\sum_{i=1}^{n}{\left({\hat{y}}_{i}-\bar{y}\right)}^{2}$ for ${\hat{y}}_{i}=X^{⊤}\hat{\beta }$ and $S_{yy}=\sum_{i=1}^{n}{\left(y_{i}-\bar{y}\right)}^{2}$. From Table 1, it is seen that ${\hat{\beta }}_{\psi }^{\left(S\right)}\left(k,d\right)$ performs better than ridge regression, since it offers smaller GCV and bigger R-squared values in the presence of multicollinearity and outliers. Moreover, because of the existence of outliers in the data set, it can be seen that R-squared’s of robust type estimators are more acceptable than the R-squared of non-robust type estimator.

**Table 1:** Evaluation of proposed estimators for the riboflavin production data set.

Estimator	$\hat{\beta }\left(k\right)$	${\hat{\beta }}_{\psi }\left(k\right)$	${\hat{\beta }}_{\psi }^{\left(S\right)}\left(k,d\right)$
CV	13.10023	9.88070	8.01023
min(GCV)	22.88144	17.00815	14.23133
$R^{2}$	0.707080	0.759485	0.798853

For further illustrative purposes, we analyze some simulated data sets in the forthcoming section.

## Monte-Carlo simulation

In this section, we perform some Monte-Carlo simulation studies to justify our assertions as well as examining the performance of the proposed estimators. As pointed and explained in Section 1, high-dimensional case *p* > *n* causes the matrix ***X***^⊤^ ***X*** to be ill-conditioned. To accommodate ill-conditioning, apart from generating multicollinear data, we will evaluate how our estimators work for the high-dimensional case *p* > *n*.

We also examine the robustness of the proposed estimators in the presence of contaminated data. The regressors are drawn a new in every replication. The efficiencies of ${\hat{\beta }}_{i}$ relative to ${\hat{\beta }}_{1}$ are defined by

$$\begin{array}{c} \text{eff}\left({\hat{\beta }}_{i},{\hat{\beta }}_{1}\right)=\frac{\frac{1}{M}\sum_{m=1}^{M}{∥{\hat{\beta }}_{1}\left(m\right)-\beta ∥}^{2}}{\frac{1}{M}\sum_{m=1}^{M}{∥{\hat{\beta }}_{i}\left(m\right)-\beta ∥}^{2}},\;i=2,3, \end{array}$$ (12)

where *M* is the number of iterations and ${\hat{\beta }}_{i}\left(m\right)$ is the *i*th estimator of ***β*** in the *m*th stage.

To examine the performance of the proposed estimators, we perform a Monte-Carlo simulation. To achieve different degrees of collinearity, following McDonald and Galarneau [23] and Gibbons [24] the explanatory variables were generated for (*n*, *p*) = {(180, 60), (180, 120)} (low-dimensional) and (*n*, *p*) = {(200, 240), (200, 360), (250, 10000)} (high-dimensional) from the following model:

$$\begin{array}{c} x_{ij}={\left(1-\gamma^{2}\right)}^{\frac{1}{2}}z_{ij}+\gamma z_{ip},\;\;i=1,2,\ldots ,n,\;j=1,2,\ldots ,p, \end{array}$$ (13)

where *z*_*ij*_ are independent standard normal pseudo-random numbers and *γ* is specified so that the correlation between any two explanatory variables is given by *γ*^2^. These variables are then standardized so that ***X***^⊤^ ***X*** and ***X***^⊤^ ***y*** are in correlation forms. Two different sets of correlation corresponding to *γ* = 0.20, 0.50, 0.90 and 0.95 are considered. The observations for the dependent variable are determined by

$$\begin{array}{c} y=X\beta +\epsilon , \end{array}$$ (14)

in sparse case: *β*_*i*_, *i* = 1, …, 0.1*p* are generated from standard normal distribution and *β*_*i*_ = 0, *i* > 0.1*p*; non-sparse case: *β*_*i*_, *i* = 1, …, 0.2 × *p* are generated from standard normal distribution and *β*_*i*_ = 0, *i* > 0.2*p*. Also, we considered $\epsilon ={\left(\epsilon_{1}^{⊤},\epsilon_{2}^{⊤}\right)}^{⊤}$ where

$$\begin{array}{ccc} & & \epsilon_{1\;(h\times 1)}∼N_{h}\left(0,\sigma^{2}V\right),\;\sigma^{2}=0.44,\;v_{ij}=\exp(-9|i-j|),\\ & & \epsilon_{2\;((n-h)\times 1)}{∼}^{{}^{\;i.i.d.}}\;\chi_{1}^{2}\left(8\right), \end{array}$$

where $\chi_{m}^{2}\left(\delta \right)$ is the non-central chi-squared distribution with *m* degrees of freedom and non-centrality parameter *δ*. The main reason of selecting such structure for errors is to contaminate the data and evaluate the robustness of the estimators. We set the first *h* error terms as dependent normal random variables and the last (*n* − *h*) error terms as independent non-central chi-squared random variables. The non-centrality causes the outliers to lie on one side of the true regression model which then pulls the non-robust estimation toward them.

The Monte-Carlo simulation is performed with *M* = 10^3^ replications, obtaining the proposed estimators ${\hat{\beta }}_{1}=\hat{\beta }\left(k\right),\;{\hat{\beta }}_{2}={\hat{\beta }}_{\psi }\left(k\right)$ and ${\hat{\beta }}_{3}={\hat{\beta }}_{\psi }^{\left(S\right)}\left(k,d\right)$, in the sparse and non-sparse regression models.

**Fig 5:**
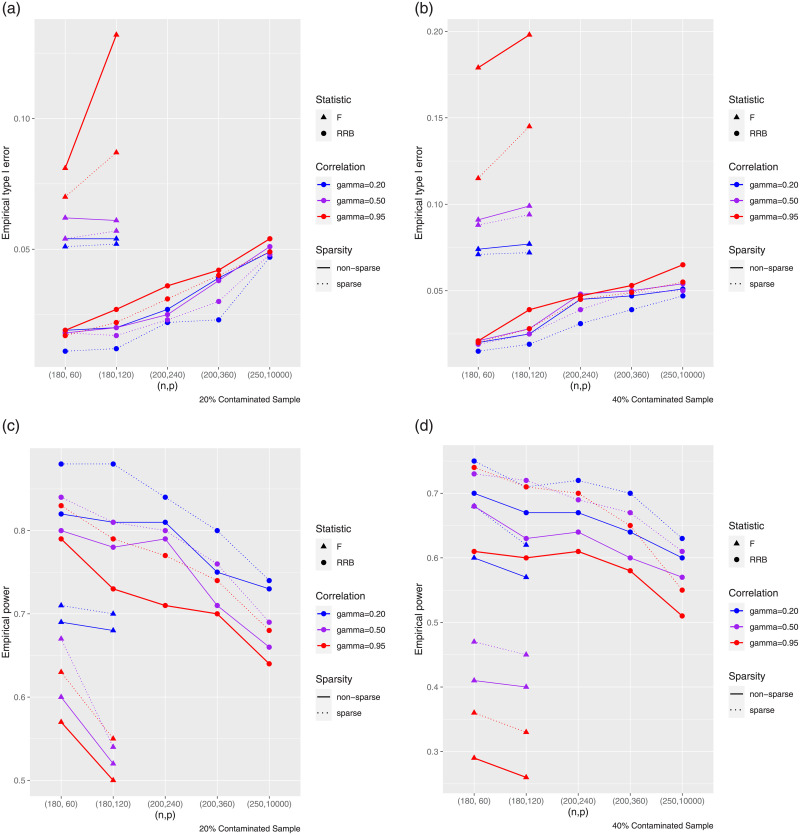
The diagram of empirical type I error and power for different (*n*, *p*) for the simulated data sets.

To save space, the Tables have been reported in the S1 File of this paper and their results have been briefly shown by Fig 5. In the “S1 File”, we provided 12 tables (S2-S9 Tables in S1 File) to extensively analyze the numerical outputs. However, to save space here, we only report Fig 5 as an abstract of tables’ results. Fig 5 summarizes the empirical type I errors and powers at a 5% significance level under low-dimensional (based on F test statistic) and high-dimensional (based on *R*_*n*_(*k*) test statistics) settings for *γ* = 0.20, 0.50, 0.90 and 0.95, respectively. The contaminated sample is the percentage of the sample contaminated with outliers ($\text{CS}=100\times \frac{n-h}{n}\%$). The F-test is valid when *p* is less than *n*. Please note in Tables S10-S13 Tables in “S1 File”, we numerically estimated the risks and efficiencies of the proposed estimators relative to ${\hat{\beta }}_{1}$.

**Fig 6:**
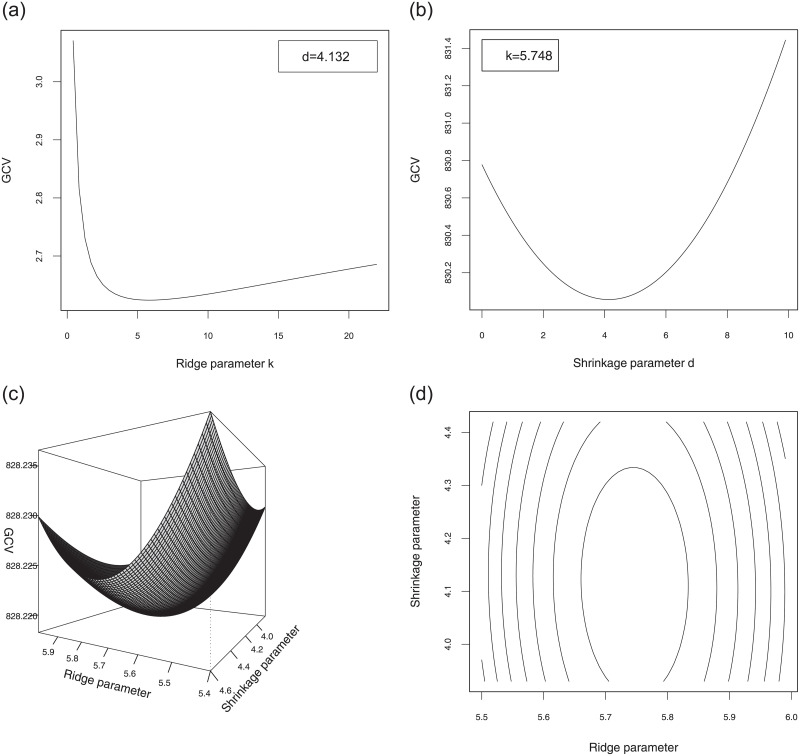
The diagram of GCV and its counter plot versus *k* and *d* for the simulated data set.

**Fig 7:**
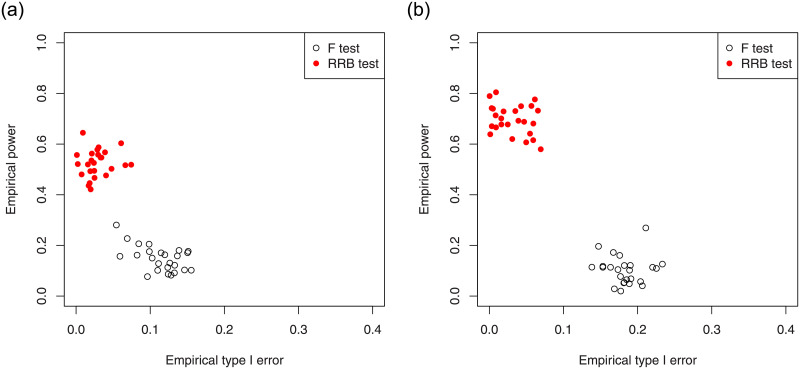
Comparison between F test and RRB test for the non-sparse case (left diagram) and sparse case (right diagram) with *γ* = 0.95, *n* = 180, *p* = 120 and *CS* = 40%.

We apply the generalized cross-validation (GCV) method to select the optimal ridge parameter (*k*_*opt*_) and shrinkage parameter (*d*_*opt*_), which minimizes the GCV function. Since the results were similar across cases, to save space we report here only the results for the sparse case with *γ* = 0.95, *n* = 200, *p* = 240 and *CS* = 20%. For this case, the minimum of GCV approximately occurred at *k*_*opt*_ = 5.90 and *d*_*opt*_ = 0.006254 for the model (14). The 3D diagram as well as the 2D slices of GCV versus *k* and *d* are plotted in Fig 6. Fig 7 shows the results of the F test and RRB test for the non-sparse and sparse cases with parameter values *γ* = 0.95, *n* = 180, *p* = 120, *CS* = 40% and significance level *α* = 5% (there are a total of 25 realizations). Each point in the plot corresponds to one realization of this configuration.

Comparison results based on the simulations are similar: Firstly, we observe the empirical type I errors for the F test, are not reasonable in comparison with the significance level *α* = 0.05, while the powers are slightly smaller than RRB test in most cases. Secondly, the RRB test is highly efficient for all cases under consideration. Its sizes are reasonable, while its powers compared to F test is high, in the low dimensional settings. Thirdly, RRB test is powerful in the high-dimensional settings, as we would expect.

As demonstrated, the RRB test is overly conservative. In other words, it achieves smaller empirical type I error and bigger empirical power compared to the F test.

## Summary & conclusions

In this paper, we proposed a robust ridge test statistic to improve the predictions in a regression model. In the presence of multicollinearity and outliers, we introduced robust ridge type estimator and improved it by shrinking toward the origin to incorporate the information contained in the null-hypothesis. By defining a generalized cross validation criterion optimal values of ridge and shrinkage parameters obtained simultaneously. Figs 3 and 6 showed the global minimum archived by this criterion. Through nonlinear minimization algorithms, we found the global minimum of this criterion with respect to both parameters. Finally, a Monte-Carlo simulation study as well as a real data example were considered to compare performances of the proposed estimators numerically.

According to Fig 5 and the detailed tabulated numerical results in the “S1 File”, we observed that the proposed robust ridge-type test statistic is more powerful than the classical F test in the presence of multicollinearity and outliers. Moreover, we found that efficiencies of robust type estimators with respect to non-robust type increase when the percentage of outliers increases. Another factor affecting the efficiency of the estimators was the number of explanatory variables. We seen the estimator ${\hat{\beta }}_{\psi }^{\left(S\right)}\left(k,d\right)$ is leading to be the best estimator among others, since it offers smaller risk and bigger efficiency values in all cases. Moreover, $\hat{\beta }\left(k\right)$ was not a suitable estimator in the presence of outliers, especially, for the high percentage of outliers. For the real examples, from Table 1, we deduced ${\hat{\beta }}_{\psi }^{\left(S\right)}\left(k,d\right)$ is quite efficient in the sense that it has significant value of goodness of fit.

## Supporting information

S1 File (PDF)

## References

[1] Lee JM, Zhang S, Saha S, Anna SS, Jiang C, Perkins J. RNA expression analysis using an antisense Bacillus subtilis genome array. J. Bacteriology. 2001; 183:7371–7380. 10.1128/JB.183.24.7371-7380.2001 PMC95586 11717296

[2] Zamboni N, Fischer E, Muffler A, Wyss M, Hohmann HP, Sauer U. Transient expression and flux changes during a shift from high to low riboflavin production in continuous cultures of Bacillus subtilis. Biotechnology and Bioengineering. 2005; 89:219–232. 10.1002/bit.20338 15584023

[3] Tibshirani R. Regression shrinkage and selection via the Lasso. J. Royal Statist. Soc. Ser. B. 1996; 58:267–288.

[4] Fan J. Li R. Variable selection via nonconcave penalized likelihood and its oracle properties. *J. Amer. Statist. Assoc*. 2001; 96:1348–1360. 10.1198/016214501753382273

[5] Zhang CH. Nearly unbiased variable selection under minimax concave penalty. *Ann. Statist*. 2010; 38:894–942. 10.1214/09-AOS729

[6] Jureckova J. Nonparametric estimate of regression coefficients. The Annals of Mathematical Statistics. 1971; 42:1328–1338.

[7] Jaeckel LA. Estimating regression coefficients by minimizing the dispersion of the residuals. The Annals of Mathematical Statistics. 1972; 43:1449–1458. 10.1214/aoms/1177692377

[8] Kibria BMG. Some Liu and Ridge Type Estimators and their Properties Under the ill-conditioned Gaussian Linear Regression Model. J. Statist. Comp. Sim. 2012; 82:1–17. 10.1080/00949655.2010.519705

[9] Tikhonov AN. Solution of incorrectly formulated problems and the regularization method. Tran. Soviet Math. 1963; 4:1035–1038.

[10] Hoerl AE, Kennard RW. Ridge regression: biased estimation for non-orthogonal problems. Thechnometrics. 1970; 12:69–82. 10.1080/00401706.1970.10488635

[11] Akdenïz F, Tabakan G. Restricted ridge estimators of the parameters in semiparametric regression model. Comm. Statist. Theo. Meth. 2009; 38:1852–1869. 10.1080/03610920802470109

[12] Roozbeh M. Robust ridge estimator in restricted semiparametric regression models. J. Mult. Anal. 2016; 147:127–144. 10.1016/j.jmva.2016.01.005

[13] Helton KH, Hjort NL. Fridge: Focused fine-tuning of ridge regression for personalized predictions. Statist. Med. 2018; 37:1290–1303. 10.1002/sim.7576 29314109

[14] Roozbeh M, Hesamian G, Akbari MG. Ridge estimation in semi-parametric regression models under the stochastic restriction and correlated elliptically contoured errors. Journal of Computational and Applied Mathematics 2020; 378. 10.1016/j.cam.2020.112940

[15] Golub G, Heath M, Wahba G. Generalized cross validationas a method for choosing a good ridge parameter. Technometrics. 1979; 21:215–223. 10.1080/00401706.1979.10489751

[16] Amini M, Roozbeh M. Optimal partial ridge estimation in restricted semiparametric regression models. J. Mult. Anal. 2015; 136:26–40. 10.1016/j.jmva.2015.01.005

[17] Speckman P. Kernel somoothing in partial linear models. J. Royal Statist Soc. Ser. B. 1988; 50:413–436.

[18] Roozbeh M, Arashi M, Hamzah NA. Generalized cross validation for simultaneous optimization of tuning parameters in ridge regression. Iranian J. Sci. Tech. Trans. A Sci. 2020; 44, 473–485. 10.1007/s40995-020-00851-1

[19] Saleh AKMdE. Theory of Preliminary Test and Stein-type Estimation with Applications, Wiley, New York; 2006.

[20] Saleh AKMdE, Arashi M, Kibria BMG. Theory of Ridge Regression Estimation with Applications, John Wiley, USA; 2019.

[21] Hettmansperger TP, McKean JW. Robust Nonparametric Statistical Methods. Second edition, Arnold: London; 2011.

[22] Roozbeh M. Optimal QR-based estimation in partially linear regression models with correlated errors using GCV criterion. Computational Statistics & Data Analysis 2018; 117:45–51. 10.1016/j.csda.2017.08.002

[23] McDonald GC, Galarneau DI. A monte carlo evaluation of some ridge-type estimators. J. Amer. Statist. Assoc. 1975; 70:407–416. 10.1080/01621459.1975.10479882

[24] Gibbons DG. A simulation study of some ridge estimators. J. Amer. Statist. Assoc. 1981; 76:131–139. 10.1080/01621459.1981.10477619

