# Supplementary material for: Ridge regression and its applications in genetic studies
Source: PLoS One. 2021 Apr 8;16(4):e0245376. doi: 10.1371/journal.pone.0245376 (PMC8031387; doi:10.1371/journal.pone.0245376)
Supplement: S1 File — (PDF) [file pone.0245376.s001.pdf]

Supplemental to "Ridge regression and its applications in genetic studies"

M. Arashi[1◑], M. Roozbeh[2*◑], N. A. Hamzah[3¤], M. Gasparini[4]

1 Department of Statistics, Faculty of Mathematical Sciences, Ferdowsi University of Mashhad, Mashhad, Iran
2 Department of Statistics, Faculty of Mathematics, Statistics and Computer Sciences, Semnan University, P.O. Box 35195-363, Semnan, Iran
3 UM Centre of Data Analytics, Institute of Mathematical Sciences, University of Malaya, Kuala Lumpur, Malaysia
4 Faculty of Mathematics, Polytechnic of Torino University, Torino, Italy

* mahdi.roozbeh@semnan.ac.ir (Corresponding Author)

Abstract

In this supplementary file, we will briefly review the theory of rank regression, some more numerical tabulation for the assessment of our proposal, and the proof of main results.

Brief Theory with an Example of Rank Regression

In this section, we briefly explain how to derive a rank estimator in the simple linear regression model. Consider the set of realizations $\{(Xi, y_i)\}_{i=1}^{n}$ from the following simple regression model

$$y_i = \alpha + \beta X_i + \epsilon_i, \quad i = 1, \dots, n, \tag{1}$$

where α and β are the intercept and slope parameters. We further assume the error components are not necessarily normal, however, they have finite Fisher information. According to the pseudo-normal (2) in the paper, define the dispersion function

$$\begin{aligned} D(\beta) &= \sum_{i=1}^{n} \epsilon_i a(R(\epsilon_i)) \\ &= \sum_{i=1}^{n} (y_i - \alpha - \beta X_i) a(R((y_i - \alpha - \beta X_i))) \\ &= \sum_{i=1}^{n} (y_i - \beta X_i) a(R((y_i - \beta X_i))), \end{aligned} \tag{2}$$

since $\sum_{i=1}^{n} a(R(y_i - \alpha - \beta X_i)) = 0$. Note that $a(i)$ is specified by the score function $\psi(\cdot)$. The scores are defined by the function $\psi(u)$, $0 < u < 1$ such that $\int_0^1 \psi(u)du = 0$ and $\int_0^1 \psi^2(u)du = 1$. We also assume that $-\psi(u) = \psi(1-u)$, that is, it is skew-symmetric about $\frac{1}{2}$. One function that satisfies the above is the Wilcoxon score as follows:

$$\psi(u) = \sqrt{12}\left(u - \frac{1}{2}\right). \tag{3}$$

S1 Table. Telephone data. The top rows are years and the bottom rows are the number of calls (No. calls).

Years	50	51	52	53	54	55	56	57	58	59	60	61
No. calls	0.44	0.47	0.47	0.59	0.66	0.73	0.81	0.88	1.06	1.20	1.35	1.49
Years	62	63	64	65	66	67	68	69	70	71	72	73
No. calls	1.61	2.12	11.90	12.40	14.20	15.90	18.20	21.20	4.30	2.40	2.70	2.90

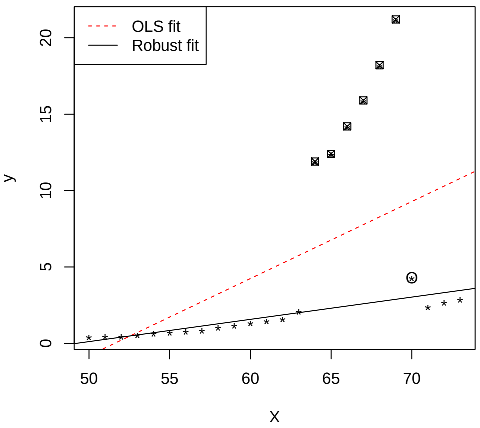

S1 Fig. Classical example demonstrating the performance of robust technique. The red dashed-dotted line is the least-squares line which is pulled toward the outlier(s). The black solid line is The Wilcoxon estimator for the telephone data.

The rank estimator $\hat{\beta}$ is the one that satisfies

$$\min_{\beta} D(\beta) = D(\hat{\beta}). \tag{4}$$

According to Hettmansperger and McKean [1], we use the following estimates for the intercept and slope parameters.

$$\begin{aligned} \hat{\alpha} &= \text{median}_{1\leq i\leq n}(y_i - X_i\hat{\beta}), \\ \hat{\beta} &= \text{median}_{1\leq i<j\leq n}\left(\frac{y_j - y_i}{X_j - X_i}\right). \end{aligned} \tag{5}$$

As a numerical example, consider the telephone data (see Table 1) from Hettmansperger and McKean [1]. The response for this data set is the number of telephone calls (tens of millions) made in Belgium for the years 1950 through 1973. Time, the years, serves as our only predictor variable. The Wilcoxon estimates of the intercept and slope are -7.13 and 0.145, respectively, while the OLS estimates are -26.006 and 0.504. The lines have been illustrated in Figure 1 in which the serious outliers have been surrounded by squares and the light outlier has been surrounded by a circle.

Detailed Numerical Assessments

In this section, we provide a more detailed analysis of the results of Monte-Carlo simulation for the assessment of the proposed estimators. Refer to the main paper for details of the simulation scheme.

S2 Table. Empirical type I error of the F-test and the proposed test (RRB) for $\mathcal{H}_o : \boldsymbol{\beta} = \mathbf{0}$ vs $\mathcal{H}_A : \boldsymbol{\beta} \neq \mathbf{0}$ at significant level 5% for $\gamma = 0\ .20$

	non-sparse case														
(n, p)	(180,60)	(180,120)	(200,240)	(200,360)	(250,10000)	(180,60)	(180,120)	(200,240)	(200,360)	(250,10000)	(180,60)	(180,120)	(200,240)	(200,360)	(250,10000)
	20% Contaminated Sample					30% Contaminated Sample					40% Contaminated Sample				
F statistic	0.054	0.054	——-	——-	——-	0.060	0.059	——-	——-	——-	0.074	0.077	——-	——-	——-
RRB statistic	0.019	0.020	0.027	0.039	0.049	0.022	0.021	0.032	0.036	0.047	0.020	0.025	0.045	0.047	0.051
	sparse case														
(n, p)	(180,60)	(180,120)	(200,240)	(200,360)	(250,10000)	(180,60)	(180,120)	(200,240)	(200,360)	(250,10000)	(180,60)	(180,120)	(200,240)	(200,360)	(250,10000)
	20% Contaminated Sample					30% Contaminated Sample					40% Contaminated Sample				
F statistic	0.051	0.052	——-	——-	——-	0.056	0.056	——-	——-	——-	0.071	0.072	——-	——-	——-
RRB statistic	0.011	0.012	0.022	0.023	0.047	0.014	0.019	0.028	0.031	0.039	0.015	0.019	0.031	0.039	0.047

S3 Table. Empirical type I error of the F-test and the proposed test (RRB) for $\mathcal{H}_o : \boldsymbol{\beta} = \mathbf{0}$ vs $\mathcal{H}_A : \boldsymbol{\beta} \neq \mathbf{0}$ at significant level 5% for $\gamma = 0\ .50$

	non-sparse case														
(n, p)	(180,60)	(180,120)	(200,240)	(200,360)	(250,10000)	(180,60)	(180,120)	(200,240)	(200,360)	(250,10000)	(180,60)	(180,120)	(200,240)	(200,360)	(250,10000)
	20% Contaminated Sample					30% Contaminated Sample					40% Contaminated Sample				
F statistic	0.062	0.061	——-	——-	——-	0.070	0.074	——-	——-	——-	0.091	0.099	——-	——-	——-
RRB statistic	0.018	0.020	0.025	0.038	0.051	0.020	0.027	0.038	0.045	0.049	0.021	0.028	0.048	0.050	0.054
	sparse case														
(n, p)	(180,60)	(180,120)	(200,240)	(200,360)	(250,10000)	(180,60)	(180,120)	(200,240)	(200,360)	(250,10000)	(180,60)	(180,120)	(200,240)	(200,360)	(250,10000)
	20% Contaminated Sample					30% Contaminated Sample					40% Contaminated Sample				
F statistic	0.054	0.057	——-	——-	——-	0.059	0.062	——-	——-	——-	0.088	0.094	——-	——-	——-
RRB statistic	0.018	0.017	0.023	0.030	0.048	0.015	0.017	0.033	0.045	0.046	0.019	0.025	0.039	0.049	0.050

S4 Table. Empirical type I error of the F-test and the proposed test (RRB) for $\mathcal{H}_o: \boldsymbol{\beta} = \mathbf{0}$ vs $\mathcal{H}_A: \boldsymbol{\beta} \neq \mathbf{0}$ at significant level 5% for $\gamma = 0\ .90$

	non-sparse case														
(n,p)	(180,60)	(180,120)	(200,240)	(200,360)	(250,10000)	(180,60)	(180,120)	(200,240)	(200,360)	(250,10000)	(180,60)	(180,120)	(200,240)	(200,360)	(250,10000)
	20% Contaminated Sample					30% Contaminated Sample					40% Contaminated Sample				
F statistic	0.078	0.105	——-	——-	——-	0.081	0.140	——-	——-	——-	0.138	0.143	——-	——-	——-
RRB statistic	0.018	0.025	0.036	0.045	0.054	0.019	0.025	0.037	0.048	0.059	0.019	0.028	0.047	0.048	0.064
	sparse case														
(n,p)	(180,60)	(180,120)	(200,240)	(200,360)	(250,10000)	(180,60)	(180,120)	(200,240)	(200,360)	(250,10000)	(180,60)	(180,120)	(200,240)	(200,360)	(250,10000)
	20% Contaminated Sample					30% Contaminated Sample					40% Contaminated Sample				
F statistic	0.062	0.085	——-	——-	——-	0.077	0.111	——-	——-	——-	0.106	0.132	——-	——-	——-
RRB statistic	0.015	0.018	0.031	0.039	0.051	0.017	0.023	0.032	0.040	0.056	0.018	0.021	0.035	0.043	0.059

S5 Table. Empirical type I error of the F-test and the proposed test (RRB) for $\mathcal{H}_o: \boldsymbol{\beta} = \mathbf{0}$ vs $\mathcal{H}_A: \boldsymbol{\beta} \neq \mathbf{0}$ at significant level 5% for $\gamma = 0\ .95$

	non-sparse case														
(n,p)	(180,60)	(180,120)	(200,240)	(200,360)	(250,10000)	(180,60)	(180,120)	(200,240)	(200,360)	(250,10000)	(180,60)	(180,120)	(200,240)	(200,360)	(250,10000)
	20% Contaminated Sample					30% Contaminated Sample					40% Contaminated Sample				
F statistic	0.081	0.132	——-	——-	——-	0.085	0.155	——-	——-	——-	0.179	0.198	——-	——-	——-
RRB statistic	0.019	0.027	0.036	0.042	0.054	0.021	0.035	0.040	0.049	0.057	0.021	0.039	0.047	0.053	0.065
	sparse case														
(n,p)	(180,60)	(180,120)	(200,240)	(200,360)	(250,10000)	(180,60)	(180,120)	(200,240)	(200,360)	(250,10000)	(180,60)	(180,120)	(200,240)	(200,360)	(250,10000)
	20% Contaminated Sample					30% Contaminated Sample					40% Contaminated Sample				
F statistic	0.070	0.087	——-	——-	——-	0.081	0.122	——-	——-	——-	0.115	0.145	——-	——-	——-
RRB statistic	0.017	0.022	0.031	0.040	0.049	0.018	0.027	0.039	0.042	0.052	0.020	0.028	0.045	0.049	0.055

S6 Table. Empirical power of the F-test and the proposed test (RRB) for $\mathcal{H}_o : \boldsymbol{\beta} = \mathbf{0}$ vs $\mathcal{H}_A : \boldsymbol{\beta} \neq \mathbf{0}$ at significant level 5% for $\gamma = 0\ .20$

	non-sparse case														
(n, p)	(180,60)	(180,120)	(200,240)	(200,360)	(250,10000)	(180,60)	(180,120)	(200,240)	(200,360)	(250,10000)	(180,60)	(180,120)	(200,240)	(200,360)	(250,10000)
	20% Contaminated Sample					30% Contaminated Sample					40% Contaminated Sample				
F statistic	0.69	0.68	——	——	——	0.64	0.60	——	——	——	0.60	0.57	——	——	——
RRB statistic	0.82	0.81	0.81	0.75	0.73	0.77	0.71	0.72	0.70	0.61	0.70	0.67	0.67	0.64	0.60
	sparse case														
(n, p)	(180,60)	(180,120)	(200,240)	(200,360)	(250,10000)	(180,60)	(180,120)	(200,240)	(200,360)	(250,10000)	(180,60)	(180,120)	(200,240)	(200,360)	(250,10000)
	20% Contaminated Sample					30% Contaminated Sample					40% Contaminated Sample				
F statistic	0.71	0.70	——	——	——	0.67	0.65	——	——	——	0.68	0.62	——	——	——
RRB statistic	0.88	0.88	0.84	0.80	0.74	0.80	0.79	0.75	0.74	0.70	0.75	0.71	0.72	0.70	0.63

S7 Table. Empirical power of the F-test and the proposed test (RRB) for $\mathcal{H}_o : \boldsymbol{\beta} = \mathbf{0}$ vs $\mathcal{H}_A : \boldsymbol{\beta} \neq \mathbf{0}$ at significant level 5% for $\gamma = 0\ .50$

	non-sparse case														
(n, p)	(180,60)	(180,120)	(200,240)	(200,360)	(250,10000)	(180,60)	(180,120)	(200,240)	(200,360)	(250,10000)	(180,60)	(180,120)	(200,240)	(200,360)	(250,10000)
	20% Contaminated Sample					30% Contaminated Sample					40% Contaminated Sample				
F statistic	0.60	0.52	——	——	——	0.51	0.46	——	——	——	0.41	0.40	——	——	——
RRB statistic	0.80	0.78	0.79	0.71	0.66	0.72	0.70	0.67	0.65	0.60	0.68	0.63	0.64	0.60	0.57
	sparse case														
(n, p)	(180,60)	(180,120)	(200,240)	(200,360)	(250,10000)	(180,60)	(180,120)	(200,240)	(200,360)	(250,10000)	(180,60)	(180,120)	(200,240)	(200,360)	(250,10000)
	20% Contaminated Sample					30% Contaminated Sample					40% Contaminated Sample				
F statistic	0.67	0.54	——	——	——	0.55	0.50	——	——	——	0.47	0.45	——	——	——
RRB statistic	0.84	0.81	0.80	0.76	0.69	0.76	0.72	0.71	0.68	0.63	0.73	0.72	0.69	0.67	0.61

S8 Table. Empirical power of the F-test and the proposed test (RRB) for $\mathcal{H}_o: \boldsymbol{\beta} = \mathbf{0}$ vs $\mathcal{H}_A: \boldsymbol{\beta} \neq \mathbf{0}$ at significant level 5% for $\gamma = 0\ .90$

			non-sparse case												
(n, p)	(180,60)	(180,120)	(200,240)	(200,360)	(250,10000)	(180,60)	(180,120)	(200,240)	(200,360)	(250,10000)	(180,60)	(180,120)	(200,240)	(200,360)	(250,10000)
	20% Contaminated Sample					30% Contaminated Sample					40% Contaminated Sample				
F statistic	0.61	0.55	—	—	—	0.49	0.45	—	—	—	0.39	0.34	—	—	—
RRB statistic	0.80	0.75	0.74	0.74	0.70	0.72	0.69	0.66	0.64	0.55	0.64	0.61	0.61	0.60	0.55

			sparse case												
(n, p)	(180,60)	(180,120)	(200,240)	(200,360)	(250,10000)	(180,60)	(180,120)	(200,240)	(200,360)	(250,10000)	(180,60)	(180,120)	(200,240)	(200,360)	(250,10000)
	20% Contaminated Sample					30% Contaminated Sample					40% Contaminated Sample				
F statistic	0.64	0.57	—	—	—	0.52	0.49	—	—	—	0.41	0.37	—	—	—
RRB statistic	0.85	0.82	0.81	0.78	0.72	0.78	0.75	0.74	0.72	0.65	0.75	0.74	0.71	0.68	0.61

S9 Table. Empirical power of the F-test and the proposed test (RRB) for $\mathcal{H}_o: \boldsymbol{\beta} = \mathbf{0}$ vs $\mathcal{H}_A: \boldsymbol{\beta} \neq \mathbf{0}$ at significant level 5% for $\gamma = 0\ .95$

			non-sparse case												
(n, p)	(180,60)	(180,120)	(200,240)	(200,360)	(250,10000)	(180,60)	(180,120)	(200,240)	(200,360)	(250,10000)	(180,60)	(180,120)	(200,240)	(200,360)	(250,10000)
	20% Contaminated Sample					30% Contaminated Sample					40% Contaminated Sample				
F statistic	0.57	0.50	—	—	—	0.37	0.35	—	—	—	0.29	0.26	—	—	—
RRB statistic	0.79	0.73	0.71	0.70	0.64	0.70	0.69	0.65	0.65	0.56	0.61	0.60	0.61	0.58	0.51

			sparse case												
(n, p)	(180,60)	(180,120)	(200,240)	(200,360)	(250,10000)	(180,60)	(180,120)	(200,240)	(200,360)	(250,10000)	(180,60)	(180,120)	(200,240)	(200,360)	(250,10000)
	20% Contaminated Sample					30% Contaminated Sample					40% Contaminated Sample				
F statistic	0.63	0.55	—	—	—	0.47	0.44	—	—	—	0.36	0.33	—	—	—
RRB statistic	0.83	0.79	0.77	0.74	0.68	0.75	0.72	0.70	0.67	0.58	0.74	0.71	0.70	0.65	0.55

S10 Table. Evaluation of the proposed estimators for $\mathcal{H}_o : \boldsymbol{\beta} = \mathbf{0}$ vs $\mathcal{H}_A : \boldsymbol{\beta} \neq \mathbf{0}$ for $\gamma = 0\ .20$

non-sparse case

(n, p)	(180,60)	(180,120)	(200,240)	(200,360)	(250,10000)	(180,60)	(180,120)	(200,240)	(200,360)	(250,10000)	(180,60)	(180,120)	(200,240)	(200,360)	(250,10000)
	20% Contaminated Sample					30% Contaminated Sample					40% Contaminated Sample				
$\hat{\mathrm{R}}(\hat{\boldsymbol{\beta}}_1; \boldsymbol{\beta})$	29.42	81.27	162.11	232.14	1490.19	40.97	90.12	184.25	281.22	1881.36	80.01	154.17	344.22	471.84	3522.61
$\hat{\mathrm{R}}(\hat{\boldsymbol{\beta}}_2; \boldsymbol{\beta})$	23.13	51.98	95.01	131.11	701.55	26.10	52.14	95.74	134.95	728.50	26.51	50.97	92.64	132.54	734.13
$\hat{\mathrm{R}}(\hat{\boldsymbol{\beta}}_3; \boldsymbol{\beta})$	22.54	48.24	88.95	118.74	689.62	22.88	48.99	90.58	123.87	675.91	22.92	47.90	91.02	120.47	682.58
$\mathrm{eff}(\hat{\boldsymbol{\beta}}_2, \hat{\boldsymbol{\beta}}_1)$	1.27	1.56	1.70	1.77	2.12	1.56	1.73	1.92	2.08	2.58	3.02	3.02	3.72	3.56	4.80
$\mathrm{eff}(\hat{\boldsymbol{\beta}}_3, \hat{\boldsymbol{\beta}}_1)$	1.31	1.68	1.82	1.96	2.16	1.79	1.84	2.03	2.27	2.78	3.49	3.21	3.78	3.92	5.16

sparse case

(n, p)	(180,60)	(180,120)	(200,240)	(200,360)	(250,10000)	(180,60)	(180,120)	(200,240)	(200,360)	(250,10000)	(180,60)	(180,120)	(200,240)	(200,360)	(250,10000)
	20% Contaminated Sample					30% Contaminated Sample					40% Contaminated Sample				
$\hat{\mathrm{R}}(\hat{\boldsymbol{\beta}}_1; \boldsymbol{\beta})$	8.62	18.93	34.88	65.87	405.93	11.87	21.47	52.57	73.80	479.19	19.35	37.21	71.45	109.61	982.73
$\hat{\mathrm{R}}(\hat{\boldsymbol{\beta}}_2; \boldsymbol{\beta})$	6.27	12.25	20.71	40.10	214.28	7.02	12.54	31.01	42.33	236.05	6.94	13.53	24.39	36.42	307.10
$\hat{\mathrm{R}}(\hat{\boldsymbol{\beta}}_3; \boldsymbol{\beta})$	6.09	11.87	20.11	37.52	209.47	6.14	11.02	27.10	30.02	211.10	6.05	11.78	18.61	28.18	222.84
$\mathrm{eff}(\hat{\boldsymbol{\beta}}_2, \hat{\boldsymbol{\beta}}_1)$	1.37	1.54	1.68	1.64	1.89	1.69	1.71	1.70	1.74	2.03	2.79	2.75	2.93	3.01	3.20
$\mathrm{eff}(\hat{\boldsymbol{\beta}}_3, \hat{\boldsymbol{\beta}}_1)$	1.41	1.59	1.73	1.76	1.94	1.93	1.95	1.94	1.99	2.27	3.20	3.16	3.84	3.89	4.41

S11 Table. Evaluation of the proposed estimators for $\mathcal{H}_o : \boldsymbol{\beta} = \mathbf{0}$ vs $\mathcal{H}_A : \boldsymbol{\beta} \neq \mathbf{0}$ for $\gamma = 0\ .50$

non-sparse case

(n, p)	(180,60)	(180,120)	(200,240)	(200,360)	(250,10000)	(180,60)	(180,120)	(200,240)	(200,360)	(250,10000)	(180,60)	(180,120)	(200,240)	(200,360)	(250,10000)
	20% Contaminated Sample					30% Contaminated Sample					40% Contaminated Sample				
$\hat{\mathrm{R}}(\hat{\boldsymbol{\beta}}_1; \boldsymbol{\beta})$	30.24	83.14	161.82	211.05	1709.66	44.01	83.68	196.52	318.84	1948.29	83.12	158.41	351.54	480.23	3789.19
$\hat{\mathrm{R}}(\hat{\boldsymbol{\beta}}_2; \boldsymbol{\beta})$	24.59	59.81	103.07	117.91	821.95	30.99	58.52	105.09	145.59	843.42	27.99	50.94	109.86	140.83	814.88
$\hat{\mathrm{R}}(\hat{\boldsymbol{\beta}}_3; \boldsymbol{\beta})$	21.00	55.06	98.67	106.59	753.15	27.68	51.65	99.25	136.84	770.08	24.74	45.65	95.79	126.71	767.04
$\mathrm{eff}(\hat{\boldsymbol{\beta}}_2, \hat{\boldsymbol{\beta}}_1)$	1.23	1.39	1.57	1.77	2.41	1.42	1.43	1.87	2.19	2.31	2.97	3.11	3.20	3.41	4.65
$\mathrm{eff}(\hat{\boldsymbol{\beta}}_3, \hat{\boldsymbol{\beta}}_1)$	1.44	1.55	1.64	1.98	2.79	1.59	1.62	1.98	2.33	2.53	3.36	3.47	3.67	3.79	4.94

sparse case

(n, p)	(180,60)	(180,120)	(200,240)	(200,360)	(250,10000)	(180,60)	(180,120)	(200,240)	(200,360)	(250,10000)	(180,60)	(180,120)	(200,240)	(200,360)	(250,10000)
	20% Contaminated Sample					30% Contaminated Sample					40% Contaminated Sample				
$\hat{\mathrm{R}}(\hat{\boldsymbol{\beta}}_1; \boldsymbol{\beta})$	8.54	20.03	39.58	61.12	418.21	12.89	21.32	53.15	73.51	520.02	20.97	43.56	90.23	116.44	1008.26
$\hat{\mathrm{R}}(\hat{\boldsymbol{\beta}}_2; \boldsymbol{\beta})$	6.28	14.01	24.58	33.38	222.45	7.45	11.91	29.36	40.84	265.31	7.94	14.62	31.77	37.32	241.21
$\hat{\mathrm{R}}(\hat{\boldsymbol{\beta}}_3; \boldsymbol{\beta})$	5.97	13.18	22.24	31.34	195.43	7.08	11.16	27.68	38.28	245.29	7.06	12.96	27.34	31.73	204.93
$\mathrm{eff}(\hat{\boldsymbol{\beta}}_2, \hat{\boldsymbol{\beta}}_1)$	1.36	1.43	1.61	1.83	1.88	1.73	1.79	1.81	1.80	1.96	2.64	2.98	2.84	3.12	4.18
$\mathrm{eff}(\hat{\boldsymbol{\beta}}_3, \hat{\boldsymbol{\beta}}_1)$	1.43	1.52	1.78	1.95	2.14	1.82	1.91	1.92	1.92	2.12	2.97	3.36	3.30	3.67	4.92

S12 Table. Evaluation of the proposed estimators for $\mathcal{H}_o : \boldsymbol{\beta} = \mathbf{0}$ vs $\mathcal{H}_A : \boldsymbol{\beta} \neq \mathbf{0}$ for $\gamma = 0\ .90$

non-sparse case

(n, p)	(180,60)	(180,120)	(200,240)	(200,360)	(250,10000)	(180,60)	(180,120)	(200,240)	(200,360)	(250,10000)	(180,60)	(180,120)	(200,240)	(200,360)	(250,10000)
	20% Contaminated Sample					30% Contaminated Sample					40% Contaminated Sample				
$\hat{R}(\hat{\boldsymbol{\beta}}_1; \boldsymbol{\beta})$	33.36	79.67	163.77	245.46	1525.36	39.36	81.90	197.76	295.59	1902.87	78.45	161.79	357.67	510.33	3894.58
$\hat{R}(\hat{\boldsymbol{\beta}}_2; \boldsymbol{\beta})$	25.66	55.05	94.10	135.99	718.23	25.02	56.21	96.88	137.32	730.50	30.95	56.79	104.25	141.14	760.33
$\hat{R}(\hat{\boldsymbol{\beta}}_3; \boldsymbol{\beta})$	23.35	52.05	90.32	123.50	675.98	23.76	47.03	96.04	125.36	682.01	26.01	49.94	99.16	130.42	702.12
$\mathrm{eff}(\hat{\boldsymbol{\beta}}_2, \hat{\boldsymbol{\beta}}_1)$	1.31	1.48	1.74	1.80	2.12	1.57	1.45	2.04	2.15	2.60	2.53	2.84	3.43	3.61	5.12
$\mathrm{eff}(\hat{\boldsymbol{\beta}}_3, \hat{\boldsymbol{\beta}}_1)$	1.44	1.53	1.81	1.98	2.25	1.65	1.74	2.05	2.35	2.79	3.01	3.23	3.60	3.91	5.55

sparse case

(n, p)	(180,60)	(180,120)	(200,240)	(200,360)	(250,10000)	(180,60)	(180,120)	(200,240)	(200,360)	(250,10000)	(180,60)	(180,120)	(200,240)	(200,360)	(250,10000)
	20% Contaminated Sample					30% Contaminated Sample					40% Contaminated Sample				
$\hat{R}(\hat{\boldsymbol{\beta}}_1; \boldsymbol{\beta})$	8.47	19.67	42.64	63.24	408.31	11.01	22.15	51.21	76.13	516.98	21.35	45.87	98.28	132.58	1154.91
$\hat{R}(\hat{\boldsymbol{\beta}}_2; \boldsymbol{\beta})$	6.02	12.14	22.44	32.78	178.54	8.41	12.32	23.97	32.89	181.45	8.69	13.01	25.14	34.51	194.24
$\hat{R}(\hat{\boldsymbol{\beta}}_3; \boldsymbol{\beta})$	5.88	10.90	22.10	27.02	165.88	7.06	12.03	21.41	30.02	167.94	7.02	12.89	22.65	29.74	193.41
$\mathrm{eff}(\hat{\boldsymbol{\beta}}_2, \hat{\boldsymbol{\beta}}_1)$	1.40	1.62	1.90	1.93	2.28	1.31	1.80	2.13	2.31	2.85	2.45	3.52	3.91	3.84	5.95
$\mathrm{eff}(\hat{\boldsymbol{\beta}}_3, \hat{\boldsymbol{\beta}}_1)$	1.41	1.80	1.93	2.34	2.46	1.56	1.84	2.39	2.53	3.08	3.04	3.56	4.34	4.48	5.97

S13 Table. Evaluation of the proposed estimators for $\mathcal{H}_o : \boldsymbol{\beta} = \mathbf{0}$ vs $\mathcal{H}_A : \boldsymbol{\beta} \neq \mathbf{0}$ for $\gamma = 0\ .95$

non-sparse case

(n, p)	(180,60)	(180,120)	(200,240)	(200,360)	(250,10000)	(180,60)	(180,120)	(200,240)	(200,360)	(250,10000)	(180,60)	(180,120)	(200,240)	(200,360)	(250,10000)
	20% Contaminated Sample					30% Contaminated Sample					40% Contaminated Sample				
$\hat{R}(\hat{\boldsymbol{\beta}}_1; \boldsymbol{\beta})$	35.97	81.45	167.87	262.20	1923.29	40.95	87.45	215.17	339.21	2078.59	89.04	194.70	401.12	512.96	4012.32
$\hat{R}(\hat{\boldsymbol{\beta}}_2; \boldsymbol{\beta})$	27.89	56.50	98.01	141.65	799.65	26.65	61.88	106.36	157.50	814.30	35.69	65.30	119.36	141.85	812.65
$\hat{R}(\hat{\boldsymbol{\beta}}_3; \boldsymbol{\beta})$	24.05	52.23	91.11	124.17	689.55	22.89	47.84	99.80	131.20	686.61	27.80	53.55	117.40	133.21	720.24
$\mathrm{eff}(\hat{\boldsymbol{\beta}}_2, \hat{\boldsymbol{\beta}}_1)$	1.29	1.44	1.71	1.85	2.41	1.53	1.41	2.02	2.15	2.55	2.49	2.98	3.36	3.62	4.94
$\mathrm{eff}(\hat{\boldsymbol{\beta}}_3, \hat{\boldsymbol{\beta}}_1)$	1.49	1.55	1.84	2.11	2.79	1.79	1.82	2.15	2.59	3.03	3.20	3.63	3.41	3.85	5.57

sparse case

(n, p)	(180,60)	(180,120)	(200,240)	(200,360)	(250,10000)	(180,60)	(180,120)	(200,240)	(200,360)	(250,10000)	(180,60)	(180,120)	(200,240)	(200,360)	(250,10000)
	20% Contaminated Sample					30% Contaminated Sample					40% Contaminated Sample				
$\hat{R}(\hat{\boldsymbol{\beta}}_1; \boldsymbol{\beta})$	10.23	26.34	46.03	64.86	454.34	12.47	22.97	56.43	80.47	531.85	24.71	45.81	109.47	139.42	1203.17
$\hat{R}(\hat{\boldsymbol{\beta}}_2; \boldsymbol{\beta})$	7.32	14.30	24.21	33.10	189.23	8.64	12.29	26.67	35.41	189.20	10.41	13.20	27.06	35.90	204.11
$\hat{R}(\hat{\boldsymbol{\beta}}_3; \boldsymbol{\beta})$	7.01	13.72	23.82	28.50	183.02	7.55	11.94	23.87	31.54	168.56	8.10	13.04	23.32	31.01	197.01
$\mathrm{eff}(\hat{\boldsymbol{\beta}}_2, \hat{\boldsymbol{\beta}}_1)$	1.40	1.84	1.90	1.96	2.40	1.44	1.87	2.11	2.27	2.81	2.37	3.47	4.05	3.88	5.89
$\mathrm{eff}(\hat{\boldsymbol{\beta}}_3, \hat{\boldsymbol{\beta}}_1)$	1.46	1.92	1.93	2.28	2.48	1.65	1.92	2.36	2.55	3.16	3.05	3.51	4.69	4.49	6.11

Proof of Main Theorems

Proof of Theorem 1. Define

$$\boldsymbol{S}(k) = (n^{-1}\boldsymbol{X}^\top \boldsymbol{X} + k\boldsymbol{I}_p)^{-1}\boldsymbol{X}^\top \boldsymbol{a}(R(\boldsymbol{y}))$$

Since for the ith component of $\boldsymbol{a}(R(\boldsymbol{\epsilon}))$, $\mathrm{E}[a(R(\epsilon_i))] = \frac{1}{n}\sum_{j=1}^{n} a(j) = 0$, we have $\mathrm{E}(\boldsymbol{S}(k)) = \boldsymbol{0}$. It is easy to see that

$$\mathrm{Var}[a(R(\epsilon_i))] = \frac{n-1}{n}\sigma_a^2, \quad \text{and} \quad \mathrm{Cov}[a(R(\epsilon_i)), a(R(\epsilon_l))] = -\frac{1}{n}\sigma_a^2.$$

Thus,

$$\begin{aligned}
\mathrm{Var}[\boldsymbol{S}(k)] &= (n^{-1}\boldsymbol{X}^\top \boldsymbol{X} + k\boldsymbol{I}_p)^{-1}\boldsymbol{X}^\top \mathrm{Var}[\boldsymbol{a}(R(\boldsymbol{y}))]\boldsymbol{X}(n^{-1}\boldsymbol{X}^\top \boldsymbol{X} + k\boldsymbol{I}_p)^{-1} \\
&= \sigma_a^2(n^{-1}\boldsymbol{X}^\top \boldsymbol{X} + k\boldsymbol{I}_p)^{-1}\boldsymbol{X}^\top \boldsymbol{X}(n^{-1}\boldsymbol{X}^\top \boldsymbol{X} + k\boldsymbol{I}_p)^{-1} \\
&= \frac{n\sigma_a^2}{k}\left\{\left(\frac{\boldsymbol{X}^\top \boldsymbol{X}}{nk} + \boldsymbol{I}_p\right)^{-1} - \left[\left(\frac{\boldsymbol{X}^\top \boldsymbol{X}}{nk} + \boldsymbol{I}_p\right)^{-1}\left(\frac{\boldsymbol{X}^\top \boldsymbol{X}}{nk} + \boldsymbol{I}_p\right)^{-1}\right]\right\}.
\end{aligned}$$

Under (A2), $\mathrm{Var}[n^{-\frac{1}{2}}\boldsymbol{S}(k)] \to \boldsymbol{\Sigma}(k)$, where

$$\begin{aligned}
\boldsymbol{\Sigma}(k) &= \frac{1}{k}\left\{\left(\frac{\boldsymbol{\Sigma}}{k} + \boldsymbol{I}_p\right)^{-1} - \left[\left(\frac{\boldsymbol{\Sigma}}{k} + \boldsymbol{I}_p\right)^{-1}\left(\frac{\boldsymbol{\Sigma}}{k} + \boldsymbol{I}_p\right)^{-1}\right]\right\} \\
&= (\boldsymbol{\Sigma} + k\boldsymbol{I}_p)^{-1} - \left[k\,(\boldsymbol{\Sigma} + k\boldsymbol{I}_p)^{-1}\,(\boldsymbol{\Sigma} + k\boldsymbol{I}_p)^{-1}\right].
\end{aligned}$$

Hence, under (A1)-(A2), using Theorem 3.5.2 of Hettmansperger and McKean (1998), we obtain $n^{-\frac{1}{2}}\boldsymbol{S}(k) \xrightarrow{\mathcal{D}} \mathcal{N}_p(\boldsymbol{0}, \boldsymbol{\Sigma}(k))$, under the null hypothesis. Thus, from the fact that $n^{-1}\boldsymbol{S}^\top(k)\boldsymbol{\Sigma}^{-1}(k)\boldsymbol{S}(k) \xrightarrow{\mathcal{D}} \chi_p^2$, we conclude that, using Slutsky's Theorem, $R_n(k) \xrightarrow{\mathcal{D}} \chi_p^2$ and the proof is complete.

Proof of Theorem 2. Using the spectral decomposition, we can write

$$\hat{\boldsymbol{\beta}}_\psi(k) = \left(\frac{1}{n}\boldsymbol{X}^\top \boldsymbol{X} + k\boldsymbol{I}_p\right)^{-1}\boldsymbol{X}^\top \hat{\boldsymbol{y}}_\psi = \boldsymbol{\Gamma}\left[\left(\frac{1}{n}\boldsymbol{\Lambda}^2 + k\boldsymbol{I}_p\right)^{-1}\boldsymbol{\Lambda}\right]\boldsymbol{\Gamma}^\top \hat{\boldsymbol{y}}_\psi, \tag{6}$$

where $\boldsymbol{\Lambda} = \mathrm{diag}(\lambda_1, \ldots, \lambda_p)$ is a diagonal matrix with eigenvalues of $\boldsymbol{X}^\top \boldsymbol{X}$ as entries with descending order and $\boldsymbol{\Gamma}$ is the respective matrix of eigenvectors. Thus we have

$$\begin{aligned}
\|\hat{\boldsymbol{\beta}}_\psi^{(S)}(k,d)\|^q &= \left\|\left(1 - \frac{d}{R_n(k)}\right)\hat{\boldsymbol{\beta}}_\psi(k)\right\|^q \\
&= \left|\left(1 - \frac{d}{R_n(k)}\right)\right|\left\|\hat{\boldsymbol{\beta}}_\psi(k)\right\|^q && (7) \\
&= \left|\left(1 - \frac{d}{R_n(k)}\right)\right|\left\|\boldsymbol{\Gamma}\left[\left(\frac{1}{n}\boldsymbol{\Lambda}^2 + k\boldsymbol{I}_p\right)^{-1}\boldsymbol{\Lambda}\right]\boldsymbol{\Gamma}^\top \hat{\boldsymbol{y}}_\psi\right\|^q && (8)
\end{aligned}$$

(i): Under the set of local alternatives $\mathcal{K}_n$, using Theorem 1, $R_n(k) > \chi_p^2(\alpha)$. Thus, from $d < 2\chi_p^2(\alpha)$, we obtain $|1 - d/R_n(k)| < 1$ for all $k > 0$ and $\alpha \in [0,1]$ values. Therefore, $\|\hat{\boldsymbol{\beta}}_\psi^{(S)}(k,d)\|^q < \left\|\hat{\boldsymbol{\beta}}_\psi(k)\right\|^q$ and $\hat{\boldsymbol{\beta}}_\psi^{(S)}(k,d)$ is a shrinkage estimator.

(ii): First note that $\|\left(\frac{1}{n}\boldsymbol{\Lambda}^2 + k\boldsymbol{I}_p\right)^{-1}\boldsymbol{\Lambda}\|^2 \leq 1$ if and only if for each i, $k > \lambda_i(1 - \lambda_i/n) = f(\lambda_i)$. It is easy to see that $f(\lambda_i)$ maximizes at the point $\lambda_i = n/2$

with the maximum value $n/2$. Thus, for $k > n/2$ we get

$$\begin{aligned}\|\hat{\boldsymbol{\beta}}_{\psi}^{(S)}(k,d)\|^q &= \left|\left(1-\frac{d}{R_n(k)}\right)\right| \left(\left\|\left[\left(\frac{1}{n}\boldsymbol{\Lambda}^2+k\boldsymbol{I}_p\right)^{-1}\boldsymbol{\Lambda}\right]\boldsymbol{\Gamma}^{\top}\hat{\boldsymbol{y}}_{\psi}\right\|^2\right)^{\frac{q}{2}} \\ &< \left|\left(1-\frac{d}{R_n(k)}\right)\right| \left(\left\|\boldsymbol{\Gamma}^{\top}\hat{\boldsymbol{y}}_{\psi}\right\|^2\right)^{\frac{q}{2}} \\ &= \left|\left(1-\frac{d}{R_n(k)}\right)\right| \left(\|\hat{\boldsymbol{y}}_{\psi}\|^2\right)^{\frac{q}{2}} \\ &= \left\|\left(1-\frac{d}{R_n(k)}\right)\hat{\boldsymbol{y}}_{\psi}\right\|^q\end{aligned}$$

and the required result follows.

(iii) The proof is similar to (ii) noting that if $k > \sup_{1\leq i\leq n}\lambda_i$, then, $k > \lambda_i = \lambda_i(1-o(n)) = \lambda_i(1-\lambda_i/n)$. The proof is complete.

References

1. Hettmansperger TP, McKean JW. Robust Nonparametric Statistical Methods. Second edition, Arnold: London; 2011.
